# Temperature Adaptive Biofilm Formation in *Yersinia enterocolitica* in Response to pYV Plasmid and Calcium

**DOI:** 10.3390/antibiotics14090857

**Published:** 2025-08-25

**Authors:** Yunah Oh, Tae-Jong Kim

**Affiliations:** Department of Forest Products and Biotechnology, Kookmin University, Seoul 02707, Republic of Korea; oya010111@kookmin.ac.kr

**Keywords:** *Yersinia enterocolitica*, biofilm formation, virulence plasmid, extracellular polymeric substances, motility, calcium, temperature adaptation

## Abstract

**Background/Objectives**: *Yersinia enterocolitica* is a pathogenic bacterium that forms biofilms, enhancing its persistence and resistance to antimicrobial agents. Biofilm formation in *Y. enterocolitica* is influenced by environmental factors such as temperature, calcium, and the presence of the virulence plasmid pYV. This study aims to explore how temperature, calcium, and pYV modulate biofilm formation in *Y. enterocolitica*, with a focus on motility and extracellular polymeric substance (EPS) production as key factors. **Methods**: *Y. enterocolitica* strains with and without the pYV plasmid were cultured at two different temperatures (26 °C and 37 °C). The effect of calcium (5 mM) on biofilm formation was tested at both temperatures. Biofilm formation was measured using crystal violet staining, motility was assessed using soft agar plates, and EPS production was quantified to determine its role in biofilm stabilization. **Results**: At 26 °C, biofilm formation increased in pYV-negative strains, driven primarily by motility and flagellar expression. In contrast, at 37 °C, pYV-positive strains showed strong biofilm formation despite reduced growth, with EPS production as the key stabilizing factor. Calcium modulated biofilm formation in a temperature-dependent manner: at 26 °C, 5 mM calcium modestly reduced biofilm formation in pYV-negative strains, while at 37 °C, it significantly suppressed both EPS production and biofilm formation by approximately 50% in pYV-positive strains. **Conclusions**: This study reveals a novel regulatory switch where temperature, calcium, and pYV modulate biofilm formation in *Y. enterocolitica*. These findings suggest that *Y. enterocolitica* can adapt between motility- and EPS-dominated biofilm strategies depending on environmental conditions. Understanding these mechanisms offers potential targets for controlling biofilm-related persistence in clinical and food safety contexts.

## 1. Introduction

*Yersinia enterocolitica* is a Gram-negative, facultative anaerobic bacterium responsible for gastroenteritis in humans. It can also lead to systemic complications such as mesenteric lymphadenitis, septicemia, or reactive arthritis, collectively referred to as yersiniosis [1]. The ability of *Y. enterocolitica* to survive and proliferate in diverse environments, including refrigerated foods and host tissues, makes it a significant environmental pathogen and a public health concern [2,3]. A critical factor contributing to *Y. enterocolitica*’s persistence in various environments is its ability to form biofilms—structured microbial communities encased in a self-produced extracellular polymeric substance (EPS) matrix [4,5]. While biofilm formation enhances environmental resilience and survival, it is not necessarily a central virulence determinant in human infections. Biofilms provide resistance to environmental stresses, antibiotics, and host immune defenses, allowing *Y. enterocolitica* to endure in diverse ecological niches. Understanding biofilm formation is essential for controlling *Y. enterocolitica* persistence in both clinical and food safety contexts.

In this study, *Y. enterocolitica* strains KT0001 and KT0003, belonging to biotype 1B and serotype O:8, were used. These strains possess distinct lipopolysaccharide (LPS) structures, which play a crucial role in their interaction with the host immune system and influence biofilm formation [6]. The LPS structure, particularly the O-antigen composition, affects bacterial surface properties and biofilm-related behaviors. Understanding the strain characteristics is critical as they directly impact biofilm phenotypes.

Several genes and regulatory pathways are known to regulate biofilm formation in *Y. enterocolitica*. These include the expression of flagella [7], the type III secretion system (T3SS) [8], and the production of extracellular polymeric substances (EPS). While the *flhDC* gene plays a key role in motility and biofilm regulation, a *flhDC* mutant and complementation strain were not included in this study due to experimental scope limitations. Future studies could benefit from including such mutants to further investigate the role of *flhDC* in biofilm regulation and motility. These factors are controlled by regulatory systems such as Rcs [9] and cyclic-di-GMP signaling [10], which contribute to biofilm architecture and its response to environmental conditions. For example, the *flhDC* operon, which regulates flagellar biosynthesis, influences biofilm formation by promoting motility at lower temperatures [11]. Moreover, the T3SS encoded by the pYV plasmid has been implicated in biofilm maturation under host-like conditions, particularly at 37 °C [12].

Environmental factors, including temperature, calcium availability, and nutrient conditions, significantly impact biofilm formation in *Y. enterocolitica*. At 26 °C, motility-driven biofilm formation predominates [7], whereas at 37 °C, EPS production and T3SS activation become more pronounced [12]. Calcium plays a key role in modulating T3SS function and biofilm stability by influencing the secretion of *Yersinia* outer proteins (Yops). Furthermore, quorum sensing and nutrient availability also play crucial roles in regulating biofilm formation. These environmental cues, along with genetic factors, determine how *Y. enterocolitica* establishes biofilms in different ecological niches.

Temperature is particularly important in regulating *Y. enterocolitica*’s behavior, as it shifts from motility-driven strategies at 26 °C to virulence-associated mechanisms at 37 °C. At ambient temperatures (26 °C), flagellar synthesis and motility drive colonization [7,13], whereas at 37 °C, mimicking the host environment, motility is suppressed, and virulence factors are activated [14]. A key genetic determinant of these adaptations is the virulence plasmid pYV, which encodes the T3SS and *Yersinia* outer proteins (Yops), facilitating immune evasion and bacterial survival during infection.

Calcium is another critical environmental factor influencing *Y. enterocolitica* physiology, particularly through its role in regulating T3SS function. A calcium-depleted environment is necessary for efficient Yop secretion, which mimics conditions encountered in intracellular host niches or the calcium-restricted gut environment [15]. The 5 mM calcium concentration used in this study was chosen because it has been shown to inhibit T3SS activation in *Y. enterocolitica* and serves as a standard high-calcium condition [16]. We observed both cell density and biofilm formation under TYE medium and 5 mM Ca^2+^ conditions and found that 5 mM Ca^2+^ treatment did not lead to significant changes in cell growth or biofilm formation. No growth inhibition or other notable physiological changes were observed, supporting the idea that the observed reduction in biofilm formation is due to calcium-specific signaling rather than osmotic stress. This concentration suppresses T3SS function and provides a physiologically relevant model for studying calcium’s regulatory effects on bacterial virulence mechanisms.

This study investigates the combined effects of temperature, the pYV plasmid, and calcium on *Y. enterocolitica* biofilm formation and associated processes, including cell growth, motility, flagellar expression, and EPS production. We hypothesize that *Y. enterocolitica* employs distinct strategies for biofilm formation depending on environmental conditions. Specifically, we aim to determine how calcium modulates these processes in relation to pYV-mediated regulation. While previous studies have examined temperature regulation of virulence and calcium-dependent T3SS activation separately, the integrated influence of these factors on biofilm development and matrix composition—especially in the context of plasmid carriage—remains poorly understood. This study fills that gap by revealing a novel regulatory switch between motility-driven and EPS-driven biofilm formation, coordinated by temperature, calcium, and the pYV plasmid.

Understanding the mechanisms regulating biofilm formation in *Y. enterocolitica* offers valuable insights into its adaptive strategies for persistence in both environmental reservoirs and host tissues. Our findings also support the development of novel antibiofilm strategies targeting temperature- or calcium-responsive virulence pathways, including EPS inhibition or T3SS regulation. These strategies could be critical for controlling *Y. enterocolitica* biofilm-mediated infections in clinical and food safety settings.

## 2. Results

### 2.1. Effects of Temperature, the Virulence Plasmid (pYV100), and Calcium on Cell Growth and Biofilm Formation

**Figure 1:**
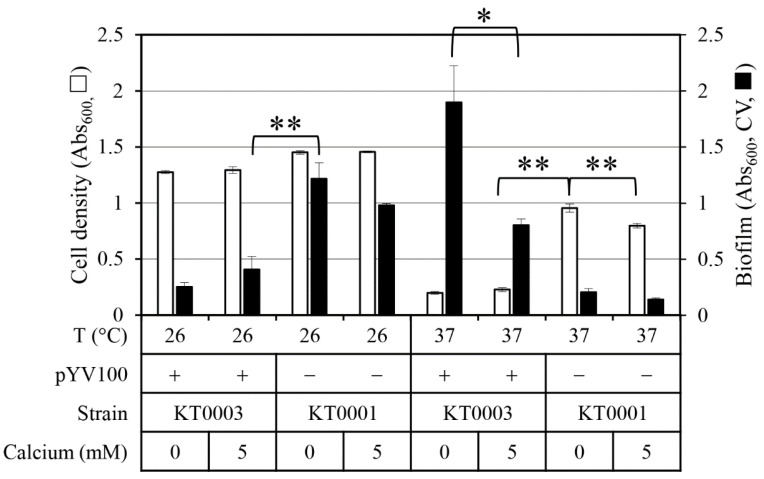
Effect of calcium concentration, virulence plasmid presence (pYV100), and temperature on cell growth and biofilm formation in *Y. enterocolitica*. Cell density (open bars, primary *Y*-axis) was measured as Abs_600_. Biofilm formation (closed bars, secondary *Y*-axis) was quantified as Abs_600_ following crystal violet (CV) staining of biomass attached to the walls of the 96-well microplates, after removal of planktonic cells. Calcium concentrations tested were 0 mM and 5 mM; temperatures tested were 26 °C and 37 °C. Data represent means ± standard deviations from three biological replicates. Statistical significance was assessed using an unpaired two-tailed Student’s *t*-test. Statistical significance is indicated by single asterisk (*) for *p* < 0.05 and double asterisks (**) for *p* < 0.01.

This study investigated the influence of the temperature, the virulence plasmid pYV100, and calcium on the growth and biofilm formation of *Y. enterocolitica* (Figure 1). At 26 °C, all cells grow well, reaching Abs_600_ values above 1.2 regardless of whether they possessed the pYV plasmid or calcium depletion, suggesting that bacterial proliferation is relatively unaffected by these factors at environmental temperatures. However, at 37 °C, strain KT0003, which possessed pYV100, exhibited significantly reduced growth, with Abs_600_ values below 0.23 regardless of the calcium concentration, while strain KT0001, which did not contain pYV100, showed only moderately reduced growth, with mean Abs_600_ values of 0.95 of at 0 mM calcium and 0.80 at 5 mM calcium. Such temperature and virulence plasmid (pYV100)-dependent growth changes are consistent with the pattern seen in a previous study [17].

At 26 °C, strain KT0001 (without pYV100) exhibited robust biofilm formation, producing an Abs_600_ value exceeding 1.0. In contrast, strain KT0003 (with pYV100) displayed significantly lower biofilm formation, producing an Abs_600_ value below 0.5. At 37 °C, the biofilm formation pattern reversed; strain KT0003 with 0 mM calcium displayed strong biofilm formation, with a mean Abs_600_ of 1.9, while strain KT0001 with 0 mM calcium produced only minimal amounts of biofilm, with Abs_600_ values below 0.2. Notably, the biofilm formation of strain KT0003 was reduced by more than half in the 5 mM calcium media.

### 2.2. Effects of Temperature, the Virulence Plasmid, and Calcium on Motility and Flagellar Expression

**Figure 2:**
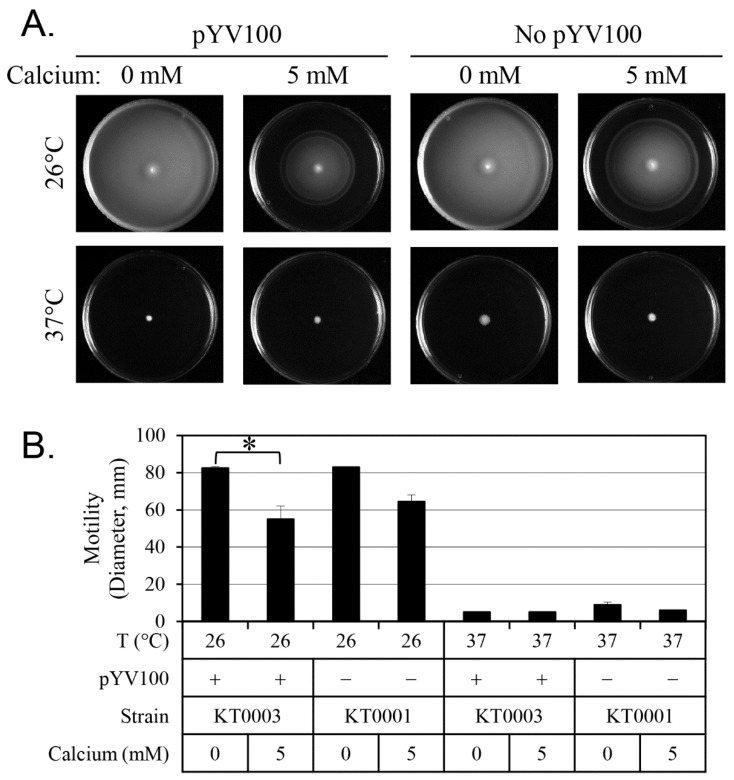
Effect of calcium concentration, pYV plasmid presence, and temperature on motility of *Y. enterocolitica*. (**A**) Representative images show radial spread from the inoculation center on soft agar plates. (**B**) Motility diameters (in mm) were measured and are presented as means ± standard deviations. Five biological replicates were analyzed per condition. Conditions tested include 0 mM and 5 mM calcium and incubation temperatures of 26 °C and 37 °C. Statistical significance was assessed using an unpaired two-tailed Student’s *t*-test. Statistical significance is indicated by single asterisk (*) for *p* < 0.05.

**Figure 3:**
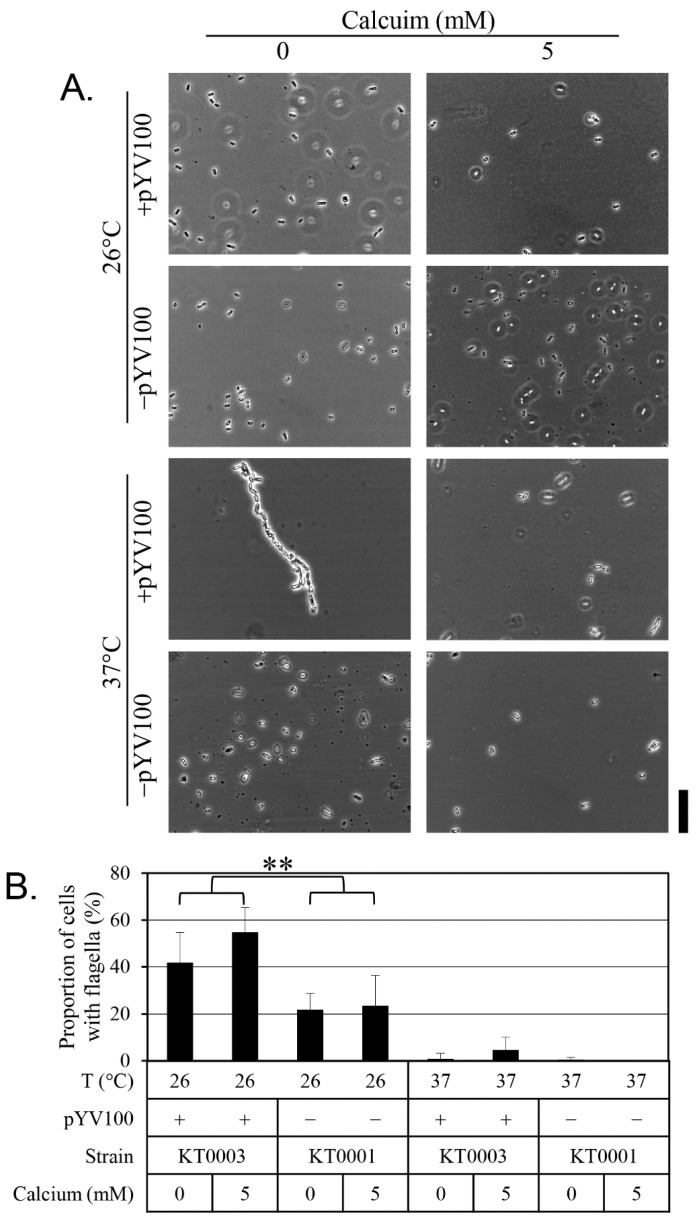
Impact of calcium, pYV plasmid presence, and temperature on flagellar expression in *Y. enterocolitica*. (**A**) Flagella were visualized by microscopy after specific staining. (**B**) Percentages of flagellated cells were quantified and are presented as means ± standard deviations from five independent experiments. Calcium concentrations tested were 0 mM and 5 mM; temperatures tested were 26 °C and 37 °C. Statistical significance was assessed using unpaired two-tailed Student’s *t*-test; *p* < 0.01 is indicated by double asterisks (**).

**Figure 4:**
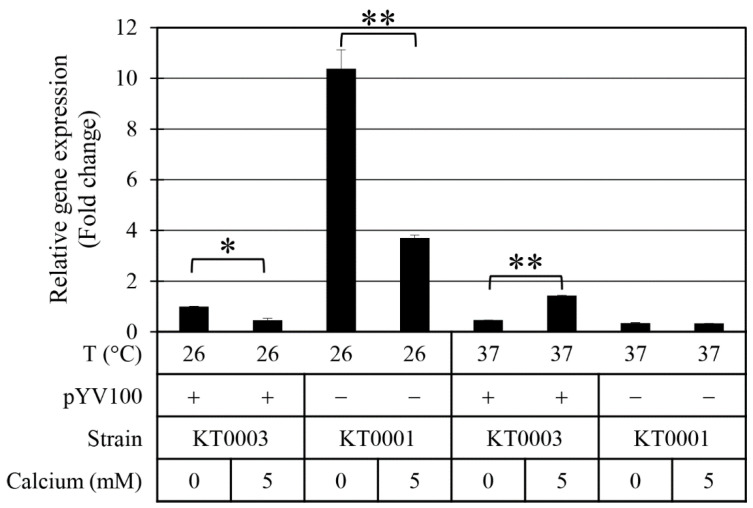
Relative expression of *flhDC*, the master regulator of flagellar synthesis, in response to calcium, pYV plasmid status, and temperature. Fold-change values were calculated by qPCR and are shown as means ± standard deviations from three independent experiments. Statistical significance was assessed using an unpaired two-tailed Student’s *t*-test. Statistical significance is indicated by single asterisk (*) for *p* < 0.05 and double asterisks (**) for *p* < 0.01.

Previous studies have suggested that in *Y. enterocolitica*, flagella actively rotate at environmental temperatures such as 26 °C and contribute significantly to biofilm formation [7]. To explore this relationship, we examined cell motility (Figure 2) and flagellar expression (Figure 3 and Figure 4) under biofilm-forming conditions to assess their correlation.

Figure 2 presents the results of the motility assays. Active swimming motility was observed at 26 °C, regardless of the presence of the virulence plasmid. In contrast, motility was nearly absent at 37 °C across all strains and conditions. Under calcium-chelated conditions (0 mM calcium), motility was most pronounced at 26 °C, with cells covering the entire Petri dish within 24 h. However, supplementation with 5 mM calcium significantly reduced motility, indicating that motility is strongly influenced by both temperature and calcium availability. Figure 3 displays the results of the flagellar expression analysis. As expected, few flagella were observed at 37 °C, while flagella were readily detected at 26 °C. Strain KT0003 exhibited a significantly higher proportion of flagellated cells than KT0001 at 26 °C (*p* < 0.01), suggesting that the presence of the virulence plasmid positively influences flagellar expression. Notably, over 40% of KT0003 cells expressed flagella in the calcium-chelated medium, and this proportion increased to over 50% in the presence of 5 mM calcium. However, calcium treatment did not significantly affect the percentage of cells with flagella, indicating that the effect of calcium on flagellar expression is relatively modest. Taken together, these findings demonstrate a clear temperature-dependent relationship between motility and flagellar expression. However, at 26 °C, no significant correlation was observed between motility and flagellar presence when considering the effects of plasmid status and calcium concentration. To quantitatively assess these relationships, Pearson correlation analyses were conducted. A moderate correlation was found between motility zone diameter and the percentage of flagellated cells at 26 °C (*r* = 0.62), but this did not reach statistical significance (*p* > 0.05). A statistically significant correlation (*r* = 0.78, *p* < 0.05) was observed between *flhDC* expression levels and biofilm formation, supporting the association shown in Figure 1.

To further investigate the molecular basis of these motility patterns, we analyzed the expression of *flhDC*—the master regulator of flagellar genes—using qPCR (Figure 4). At 26 °C, *flhDC* expression was significantly higher in strain KT0001 than in KT0003, particularly under calcium-chelated conditions, where *flhDC* transcript levels in KT0001 were more than 10.4-fold greater than in KT0003. This difference decreased to 3.7-fold when 5 mM calcium was added. At 37 °C, KT0003 showed a 43% increase in *flhDC* expression in the calcium-supplemented condition. Interestingly, a discrepancy was observed: at 26 °C, *flhDC* expression in KT0003 was relatively low in the presence of 5 mM calcium, yet flagellar presence was highest under this condition (Figure 3). This suggests the involvement of post-transcriptional regulation or additional unknown mechanisms influencing flagellar assembly beyond the transcriptional level governed by FlhDC. Further studies are required to elucidate these mechanisms. To better understand these relationships, we performed additional Pearson correlation analyses. A strong positive correlation (*r* = 0.81) was observed between *flhDC* expression and motility, while the correlation between *flhDC* expression and biofilm formation was moderate (*r* = 0.59). These results indicate that motility is likely to contribute to the biofilm phenotype at 26 °C, though it may not be the sole determining factor.

### 2.3. Effect of Temperature, the pYV Plasmid, and Calcium on Extracellular Polymeric Substance Production

**Figure 5:**
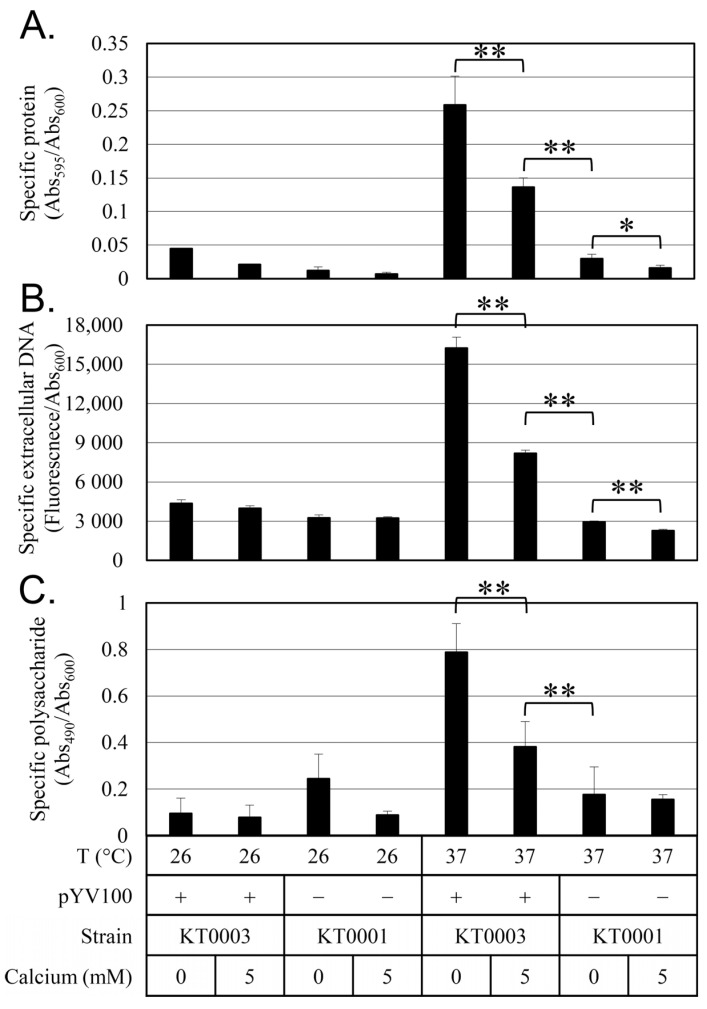
Effect of calcium, pYV plasmid status, and temperature on extracellular polymeric substance (EPS) production in *Y. enterocolitica*. (**A**) Protein content measured by Coomassie blue absorbance at 595 nm (Abs_595_). (**B**) Extracellular DNA content assessed by fluorescence at 528 nm using SYTOX™ Green dye. (**C**) Polysaccharide content measured by phenol-sulfuric acid assay at 490 nm (Abs_490_). All EPS component levels were normalized to cell density (Abs_600_). Conditions included 0 mM and 5 mM calcium and incubation at 26 °C or 37 °C. Data represent means ± standard deviations from four biological replicates. Statistical significance was assessed using an unpaired two-tailed Student’s *t*-test. Statistical significance is indicated by single asterisk (*) for *p* < 0.05 and double asterisks (**) for *p* < 0.01.

To investigate the role of EPSs in *Y. enterocolitica* biofilm formation, EPS production was analyzed in relation to temperature, the presence of the virulence plasmid, and calcium availability (Figure 5). At 26 °C, the production of EPSs—including proteins, polysaccharides, and eDNA—was limited. At 37 °C, EPS production remained low in strain KT0001, but the production of all three EPS components increased dramatically in strain KT0003, though their levels were reduced by approximately 50% in the presence of 5 mM calcium. These results show that EPS production is tightly regulated by temperature, the virulence plasmid, and calcium availability, suggesting a coordinated mechanism that integrates environmental cues with biofilm formation and virulence strategies.

## 3. Discussion

This study provides new insights into how temperature and calcium co-regulate biofilm formation in a virulence plasmid-dependent manner. Importantly, our unique contribution lies in showing that *Y. enterocolitica* regulates biofilm formation differently depending on environmental temperature. At 26 °C, representing natural environmental conditions, biofilm formation is primarily motility-driven and largely independent of T3SS activity. In contrast, at 37 °C, mimicking the host infection environment, calcium modulates EPS-driven biofilm formation through pYV-dependent T3SS regulation. This temperature-dependent dual strategy distinguishes our work from previous studies and highlights the adaptive versatility of *Y. enterocolitica* in both environmental and host settings.

By comparing pYV-positive and -negative strains, we identified a regulatory shift: in pYV-positive cells, elevated temperature and calcium limitation suppress motility while promoting EPS-driven biofilm maturation—a likely adaptation for host colonization. In contrast, pYV-negative strains maintain a motility-dominant strategy under the same conditions, suggesting alternative survival tactics shaped by plasmid carriage.

At 26 °C, biofilm formation was highest in strain KT0001 and appeared independent of T3SS activity, regardless of calcium levels. This supports previous findings that biofilm development under environmental conditions is primarily driven by flagella-mediated surface attachment [7]. In contrast, at 37 °C, the pYV-positive strain showed significantly enhanced biofilm formation under calcium-deficient conditions, consistent with the known activation of T3SS under host-mimicking conditions [18]. Calcium supplementation suppressed this phenotype, likely by inhibiting Yop secretion. These findings suggest a context-specific biofilm strategy, wherein EPS-dependent stabilization dominates under host-like conditions.

Interestingly, in the pYV-negative strain at 37 °C, both motility and biofilm formation were reduced, indicating that, in the absence of the plasmid, the bacterium lacks the capacity to produce compensatory EPS. This observation supports a dual-regulatory model in which biofilm formation requires either flagellar motility (at 26 °C) or pYV-dependent EPS production (at 37 °C), but not both simultaneously. Temperature also plays a central role in modulating motility. The pronounced reduction in swimming activity at 37 °C aligns with earlier reports showing transcriptional repression of flagellar genes at host temperature [19]. Flagella are antigenic and energetically costly; therefore, their expression may be downregulated during host infection. Consistent with this, both pYV-positive and -negative strains exhibited minimal flagellar gene expression at 37 °C (Figure 3B), reinforcing the concept of temperature-sensitive repression of motility genes. These data collectively support a model in which motility is prioritized at environmental temperatures and downregulated at host temperatures, where virulence factors such as T3SS take precedence.

Beyond FlhDC, other regulatory systems likely contribute to the observed phenotypes. One candidate is the Rcs phosphorelay system, which responds to envelope stress and regulates exopolysaccharide synthesis in *Yersinia* and other Gram-negative bacteria [20]. Cyclic-di-GMP is another global regulator that orchestrates the switch between motility and sessility [21]. Although c-di-GMP was not directly measured in this study, the temperature- and plasmid-dependent changes in biofilm architecture suggest its possible involvement. Moreover, it remains unclear whether virulence regulators such as VirF directly influence matrix gene expression, particularly under calcium-limited conditions at 37 °C. Further studies using transcriptomics and regulatory mutants will be needed to investigate these pathways.

The observed differences in EPS production between 26 °C and 37 °C further highlight the adaptive flexibility of *Y. enterocolitica*. EPS production was minimal in both strains at 26 °C, supporting the idea that motility dominates biofilm formation under environmental conditions. At 37 °C, EPS synthesis increased significantly in the pYV-positive strain under calcium-limited conditions, correlating with enhanced biofilm formation. This supports the hypothesis that EPS production is promoted by T3SS activation and is essential for biofilm stabilization under host-like conditions. However, the molecular links between T3SS activity and EPS gene expression remain unclear. It is plausible that T3SS signaling influences global regulators such as Rcs or c-di-GMP, leading to coordinated EPS upregulation.

Extracellular polymeric substances are known to shield bacteria from immune responses, including phagocytosis and antimicrobial peptides [22]. Our results suggest that calcium deficiency may enhance EPS synthesis via T3SS activation, contributing to immune evasion. The exceptionally high EPS production in pYV-positive strains under calcium-limited conditions supports a functional connection between Yop secretion and EPS biosynthesis. Furthermore, calcium ions may stabilize the Yop secretion system and thereby indirectly influence biofilm architecture—consistent with earlier studies that demonstrated calcium-dependent regulation of Yop activity and function. Importantly, although our current data provide indirect evidence, we propose potential mechanisms whereby T3SS activity could influence EPS synthesis. One possibility is that T3SS activation modulates global transcriptional regulators such as the Rcs phosphorelay system or cyclic-di-GMP signaling, both of which are known to orchestrate exopolysaccharide biosynthesis and biofilm maturation in Gram-negative bacteria [20,21]. Another possibility is post-transcriptional regulation, in which secretion-associated chaperones or small RNAs may stabilize or destabilize EPS-related transcripts. While further experimental validation is required, we believe these hypotheses provide a mechanistic framework to guide future investigations.

While our study provides robust endpoint comparisons across experimental variables, it is limited by its parallel design, which does not capture temporal changes in regulatory events or biofilm maturation. Mechanistic progressions, such as the sequential activation of matrix components or EPS dynamics, were not directly observed. A longitudinal experimental design—incorporating time-course analyses of gene expression, EPS composition, and T3SS/Yop activity—will be essential to uncover the regulatory hierarchy over time. We acknowledge this limitation and suggest that future studies incorporate such approaches to resolve stage-specific regulatory transitions.

Additionally, the antimicrobial effects of calcium itself were not addressed. Determining the minimum inhibitory concentration (MIC) of calcium for *Y. enterocolitica*, and testing sub-MIC, would help clarify whether calcium affects biofilm formation through physiological stress or regulatory signaling. Another limitation is the absence of an empty vector control. Because pYV100 is a mobilizable derivative of the naturally occurring virulence plasmid pYV8081 rather than a recombinant plasmid constructed with a cloning backbone, there is no equivalent empty vector available for this system. Therefore, we relied on comparisons between plasmid-cured and plasmid-carrying strains to evaluate phenotypic differences. Future studies may consider constructing artificial vector-based systems if backbone-specific control is required. This would be particularly relevant for assessing the use of calcium-modulating strategies in food or clinical settings.

Finally, pH was not monitored or controlled in our assays. While the TYE medium is expected to remain near neutral, we acknowledge that local pH shifts during bacterial growth could influence gene expression, motility, or matrix synthesis. Future work should include pH-controlled conditions to clarify its role in biofilm regulation.

In summary, this study identifies a novel plasmid-dependent regulatory switch in *Y. enterocolitica* biofilm development—shifting from flagella-driven colonization at 26 °C to T3SS- and EPS-dominated biofilms at 37 °C. This dual-strategy model has not been previously reported and offers new insight into how environmental and genetic factors shape virulence and persistence. The integration of temperature, calcium signaling, and pYV plasmid function defines a complex regulatory network that balances motility, T3SS activation, and matrix production in response to niche-specific demands. These findings advance our understanding of *Y. enterocolitica* pathogenesis and suggest that targeting EPS synthesis or T3SS activity may offer effective strategies to mitigate biofilm-mediated infections.

## 4. Materials and Methods

### 4.1. Strains and Culture Conditions

The bacterial strains used in this study are listed in Table 1. Strain KT0001 (*Yersinia enterocolitica* KCCM 41657) is a plasmid-cured variant lacking the virulence plasmid pYV. To obtain the nalidixic acid-resistant mutant strain KT0002, the plasmid-cured parental strain KT0001 was cultured in 5 mL of tryptone-yeast extract (TYE) medium at 26 °C for 48 h. After incubation, cells were streaked onto TYE agar plates containing 25 μg/mL nalidixic acid. If no colonies appeared, the culture was subjected to an additional round of incubation in fresh 5 mL TYE medium under the same conditions, followed by re-plating on selective agar. This subculturing process was repeated until colonies capable of growing on the selective plates were observed. Colonies that emerged on nalidixic acid-containing plates were isolated and subjected to 16S rRNA gene sequencing to confirm their identity as *Y. enterocolitica*. The resulting strain was designated KT0002. Strain KT0003 was generated by introducing pYV100 into KT0002 via a triparental mating procedure. For this, *E. coli* HB101/pYV100 (streptomycin^R^/spectinomycin^R^) served as the donor, *E. coli* DH5α/pRK2013 (kanamycin^R^) as the helper, and KT0002 (nalidixic acid^R^) as the recipient strain. The three cultures were combined with MgSO_4_, passed through a sterile filter, and incubated on LB agar for 3 h at 26 °C. After washing, cells were plated on LB agar containing streptomycin (15 μg/mL), spectinomycin (15 μg/mL), and nalidixic acid (25 μg/mL) to select for transconjugants. Colonies were screened for kanamycin sensitivity to confirm loss of the helper plasmid, and the presence of functional pYV100 was confirmed by detecting Yops secretion under Mg-oxalate culture conditions. pYV (plasmid of *Yersinia* virulence) generally refers to the family of virulence plasmids that encode the T3SS responsible for secreting Yops. In this study, pYV100 denotes the specific artificial derivative of pYV that was reintroduced into the plasmid-cured strain KT0002 to restore virulence capacity. Hereafter, we use pYV to describe the plasmid in general, and pYV100 only when referring specifically to this engineered derivative.

All strains were cultured in TYE medium, consisting of 1% tryptone (catalog number: 211705, Becton Dickinson Korea Co., Ltd., Seoul, Republic of Korea) and 0.5% yeast extract (catalog number: 2509110, Becton Dickinson Korea Co., Ltd.) dissolved in distilled water. For preparation of TYE agar plates, 1.5% Bacto agar (catalog number: 214010, Becton Dickinson Korea Co., Ltd.) was added to the medium before autoclaving.

Strains KT0001 and KT0003 were streaked onto TYE agar plates and incubated at 26 °C for 48 h. Single colonies were then inoculated into liquid TYE medium and grown overnight at 26 °C. These overnight cultures were subcultured into fresh TYE medium at an initial optical density of Abs_600_ = 0.05 and used for all subsequent experiments. All bacterial cultures for assays were incubated in 96-well microplates (SPL Life Sciences, Pocheon, Republic of Korea).

To investigate the effect of calcium, two media conditions were prepared: calcium-depleted (0 mM) and calcium-supplemented (5 mM). Cell growth was not inhibited under the 5 mM calcium condition when compared to the calcium-depleted medium (TYE). Furthermore, previous studies, such as that by DeBord et al. [16], have used 5 mM calcium to inhibit Type III secretion in *Y. enterocolitica*, suggesting that the observed changes in biofilm formation in our study are due to specific regulatory effects rather than physiological stress. For the 0 mM calcium condition, calcium ions were removed by adding 0.8 mL of 2 M MgCl_2_ and 8 mL of 0.2 M sodium oxalate to 91 mL of TYE medium. This mixture was incubated at room temperature for 24 h prior to use. For the 5 mM calcium condition, calcium chloride (CaCl_2_) was added directly to the TYE medium. The pH of the TYE medium was not specifically monitored or adjusted during the experiments. It was assumed to remain near neutral under the experimental conditions at both 26 °C and 37 °C.

### 4.2. Cell Density and Biofilm Quantification

Subcultures of each strain (in TYE media at an Abs_600_ of 0.05) were incubated at either 26 °C or 37 °C for 24 h, and then cell density and biofilm formation were measured. Three independent biological replicates were performed for each strain to ensure reproducibility. Cell density was determined by measuring the absorbance at 600 nm. Biofilm formation was quantified using a modified version of the 1% crystal violet assay described in a previous study [24], measuring only biomass that was firmly attached to the walls and bottom surfaces of the 96-well microplates. Planktonic (non-adherent) cells were removed during the washing steps and were not included in the quantification. Briefly, the cultures in the 96-well plates were washed three times with distilled water. Then, 100 μL of 1% crystal violet solution was added to each well, and the plates were incubated at room temperature for 15 min. After staining, the wells were again washed three times with distilled water, and the remaining crystal violet, which was bound in the biofilms, was solubilized by adding 100 μL of 95% ethanol. After incubating at room temperature for another 15 min, the cultures’ Abs_600_ values were measured using a Synergy™ LX Multi-Mode Reader (BioTek Instruments Korea Ltd., Seoul, Republic of Korea).

### 4.3. Cell Motility Measurement

Motility was measured using TYE agar plates containing 0.3% agar using a procedure adapted from a previous study [7]. Cells from a single colony on a TYE plate were inoculated onto the center of a plate containing 0.3% agar, and the plate was incubated at 26 °C or 37 °C for 24 h. After incubation, images of the plates were captured using a Gel Doc™ XR+ (Bio-Rad Laboratories Inc., Hercules, CA, USA), and the diameter of the motility zone was measured using the Image Lab™ software version 3.0 (Bio-Rad Laboratories Inc.).

### 4.4. Flagellar Staining

The presence and distribution of flagella were observed via flagellar staining using a method modified from a previous study [7]. The staining solution was prepared immediately before use by mixing 10 parts of mordant solution (2 g of tannic acid, 10 mL of 5% phenol, and 10 mL of saturated aqueous AlKO_8_S_2_·12H_2_O) with 1 part of dye solution (12% crystal violet in ethanol). Three μL bacterial culture samples were placed on a 50 × 24 mm glass slip and covered with a cover slip. Ten μL of staining solution was added to the edge of the coverslip to be drawn into the sample by capillary action. Flagella were then observed under an AXIO Scope.A1 microscope (ZEISS Korea, Seoul, Republic of Korea).

The percentage of flagella-forming cells was calculated according to a previous study [25]. All cells and cells with flagella within a certain area of microscope field were counted, and if necessary, additional areas were observed the same way until 100 cells were counted. The number of flagellated cells was divided by the total number of cells and multiplied by 100 to obtain the percentage of flagellated cells.

### 4.5. Measuring flhDC Expression Level Using Real-Time Polymerase Chain Reaction (qPCR)

The cells were prepared by inoculating the strains, as described in Figure 4, and incubating them for 24 h under the specified conditions. Total RNA was extracted using the AccuPrep^®^ Bacterial RNA Extraction Kit (Catalog number: K-3142, Bioneer Co., Daejeon, Republic of Korea) according to the manufacturer’s instructions. The amount of total cDNA was adjusted to a range of 1 ng to 5 μg based on the Abs_260_/Abs_280_ ratio using a GENESYS 150 UV-Vis spectrophotometer (Catalog number: 840-300300, ThermoFisher Scientific Korea Inc., Seoul, Republic of Korea). Then, complementary DNA (cDNA) synthesis was performed using the SuPrimeScript cDNA Synthesis Kit (Catalog number: SRK-1000, Genetbio Co., Ltd., Daejeon, Republic of Korea). The mRNA transcripts of *flhDC* were quantitatively analyzed using PowerUp™ SYBR™ Green Master Mix for qPCR (Catalog number: A25742, ThermoFisher Scientific Korea Inc.) in QuantStudio 5 Real-Time PCR System (Catalog number: A28574, ThermoFisher Scientific Korea Inc.) and the primer pair detailed in Table 1. The thermocycler protocol began at 50 °C for 2 min, followed by initial denaturation at 95 °C for 2 min, and then 40 cycles of 95 °C for 15 s, 59 °C for 15 s, and 72 °C for 1 min. The qPCR product size for the *flhDC* gene was 176 base pairs, and for the 16S rRNA gene, it was 183 base pairs. To confirm the quality of the amplified products, a melt curve analysis was performed at 95 °C for 15 s, followed by 60 °C for 1 min, and then a gradual temperature increases of 1.6 °C per second until reaching 95 °C, which was maintained for 15 s. The reference control for calculating relative gene expression was strain KT0003 (pYV^+^) cultured at 26 °C under calcium-free (0 mM) conditions. RNA was harvested at 24 h post-inoculation, corresponding to the same timepoint used for cell growth and biofilm quantification assays. The cycle threshold (Ct) values for the *flhDC* gene were calculated, and the 16S rRNA gene was used as the housekeeping gene for normalization. Relative gene expression levels were analyzed using the 2^−ΔΔCt^ method. Raw Ct values and replicate data are provided in Supplementary Table S1 to allow readers to evaluate variability across biological replicates.

### 4.6. Extracellular Polymeric Substance Quantification

The production of EPSs was measured to evaluate the major biofilm components: proteins, polysaccharides, and extracellular DNA (eDNA). We used a previously reported method [26] with some modifications. Strains were cultured as described in the Section 4.2. To ensure that EPS from both planktonic cells and surface-attached biofilms was included, the biofilm biomass adhering to the walls of the 96-well plates was gently scraped and suspended into the culture medium prior to centrifugation. Then, 1 mL of this mixture (containing both planktonic and detached biofilm cells) was centrifuged at 9279× *g* for 10 min to obtain a pellet and a cell-free culture supernatant. The pellet was washed once with phosphate-buffered saline, centrifuged at 9279× *g* for 10 min, and resuspended in 1 mL of isotonic buffer. The resuspended solution was incubated at 4 °C for 12 h. Then, after vortexing for 3 min, the solution was centrifuged at 9279× *g* for 15 min, and the supernatant was combined with the previously obtained cell-free cultures. This mixture was treated with 3 times its volume of iced ethanol and incubated at 20 °C for 12 h to precipitate the EPSs. The EPSs were then pelleted by centrifugation at 9279× *g* for 15 min, dried, and stored at 4 °C. The dried EPS was dissolved in distilled water for further analyses. For protein quantification, 100 μL of EPS sample was mixed with 1 mL of Bradford reagent (Biosesang, Yongin, Republic of Korea) and vortexed. Then, the mixture was incubated at room temperature for 2 min, and its Abs_595_ was measured. For polysaccharide quantification, a phenol–sulfuric acid method was used [26]. Fifty μL of EPS sample was mixed with 150 μL of sulfuric acid, followed by 30 μL of 5% phenol. The mixture was incubated at room temperature for 10 min, and its Abs_490_ was measured. For eDNA quantification, SYTOX™ Green Nucleic Acid Stain (Catalog number: S7020, ThermoFisher Scientific Korea Inc.) was used according to the manufacturer’s protocol. Fifty μL of EPS sample was mixed with 50 μL of 5 μM SYTOX™ Green Stain solution. After incubating at room temperature for 40 min, fluorescence was measured with excitation at 485 nm and measurement at 528 nm. For all three EPS constituent analyses, the measured absorbances were divided by the Abs_600_ of the corresponding culture to calculate the relative EPS production per unit cell.

### 4.7. Statistical Analysis

All data were derived from three independent biological replicates unless otherwise specified. Statistical significance between experimental groups was assessed using unpaired two-tailed Student’s *t*-tests, with *p*-values less than 0.05 considered statistically significant. Results are presented as mean ± standard deviation (SD). Statistically significant differences are indicated in the figures using either asterisks (* for *p* < 0.05 and ** for *p* < 0.01).

## 5. Conclusions

This study demonstrates that biofilm formation in *Y. enterocolitica* is regulated by a combination of environmental factors—temperature and calcium—and the presence of the pYV virulence plasmid. At 26 °C, biofilm development is primarily driven by flagella-mediated motility, especially in pYV-negative strains. In contrast, at 37 °C, the presence of pYV promotes EPS-mediated biofilm formation despite reduced bacterial growth, reflecting a temperature-dependent regulatory shift that supports adaptation to both environmental and host-associated niches. Calcium further modulates these processes in a temperature- and plasmid-dependent manner. It enhances EPS production and biofilm formation in pYV-positive strains at 37 °C, while suppressing biofilm formation at 26 °C. These findings support a regulatory framework in which environmental cues are integrated through the pYV plasmid—potentially involving the Rcs phosphorelay system or cyclic-di-GMP signaling—to coordinate biofilm architecture. Targeting EPS biosynthesis or disrupting calcium-responsive regulatory pathways may offer promising strategies for controlling *Y. enterocolitica* persistence in both clinical and food safety contexts.

## Figures and Tables

**Table 1:** Bacterial strain, plasmid, and primer information.

Name	Description	Source/ Reference
*Yersinia enterocolitica*		
KT0001	biotype 1B, serotype O:8, Sal/Esc-Ptz-CRMOX-	KCCM 41657
KT0002	KCCM 41657, Nal^R^	In this study
KT0003	KT0002 with pYV100, Nal^R^	In this study
Plasmid		
pYV100	*mob*^+^, mobilizable derivative of pYV8081, Str/Spc^R^	[23]
Primers		
*flhDC*-F	CCTCAGCGATGTTTCGTCTC	[20]
*flhDC*-R	CTGCAAGTCATCCACACGAG	[20]
16S rRNA-F	GCACGTAATGGTGGGAACTC	[20]
16S rRNA-R	CTCCAATCCGGACTACGACA	[20]

## Data Availability

The original contributions presented in this study are included in the article/Supplementary Materials. Further inquiries can be directed to the corresponding author.

## Supplementary Materials

The following supporting information can be downloaded at: https://www.mdpi.com/article/10.3390/antibiotics14090857/s1, Table S1: RT-qPCR raw Ct values of *flhDC* and housekeeping gene (16S rRNA) in *Yersinia enterocolitica.*

## Abbreviations

The following abbreviations are used in this manuscript:

EPS	Extracellular polymeric substance
T3SS	Type III secretion system
Yops	*Yersinia* outer proteins
TYE	Tryptone-yeast extract
eDNA	Extracellular DNA
SD	Standard deviation
MIC	Minimum inhibitory concentration
