# Supplementary material for: Temperature Adaptive Biofilm Formation in Yersinia enterocolitica in Response to pYV Plasmid and Calcium
Source: Antibiotics (Basel). 2025 Aug 25;14(9):857. doi: 10.3390/antibiotics14090857 (PMC12466685; doi:10.3390/antibiotics14090857)
Supplement: Supplementary file 1 [file antibiotics-14-00857-s001.zip › antibiotics-3821577-supplementary.pdf]

Supplementary Table S1. RT-qPCR raw Ct values of *flhDC* and housekeeping gene (16S rRNA) in *Yersinia enterocolitica*. Raw Ct values of *flhDC* and 16S rRNA (housekeeping gene) in *Yersinia enterocolitica* under different temperature, calcium, and plasmid conditions. Values represent individual biological replicates, with mean values calculated across three replicates. Cultures were harvested 24 h post-inoculation. Relative expression values (main text, Figure 4) were calculated using the $2^{-\Delta\Delta Ct}$ method, with KT0003 (pYV^{+}, 26 °C, 0 mM Ca^{2+}) as the reference condition.

Cell culture condition									
	Incubation temperature	26℃	26℃	26℃	26℃	37℃	37℃	37℃	37℃
	Calcium concentration	0 mM	5 mM	0 mM	5 mM	0 mM	5 mM	0 mM	5 mM
	Presence of pYV100	+	+	-	-	+	+	-	-
16S rRNA		11.794	10.896	12.745	11.928	11.857	12.372	11.034	10.463
		11.823	10.869	12.726	11.768	11.524	12.159	10.827	10.352
		11.883	10.854	12.822	11.692	11.403	12.426	10.728	10.402
Average		11.833	10.873	12.764	11.796	11.595	12.319	10.863	10.406
flhDC		20.214	20.721	17.763	18.298	21.103	20.225	20.749	20.371
		20.222	20.153	17.93	18.372	21.13	20.179	20.732	20.382
		20.274	20.326	17.741	18.261	21.102	20.22	20.718	20.417
ΔCt for *flhDC*		8.381	9.848	4.999	6.502	9.508	7.906	9.886	9.965
		8.389	9.280	5.166	6.576	9.535	7.860	9.869	9.976
		8.441	9.453	4.919	6.465	9.507	7.901	9.990	10.011
Average of ΔCt for *flhDC*		8.403	9.527	5.028	6.514	9.517	7.889	9.915	9.984
Standard deviation of ΔCt for *flhDC*		0.027	0.238	0.103	0.046	0.013	0.021	0.053	0.020
ΔΔCt for *flhDC*		-0.023	1.445	-3.405	-1.901	1.105	-0.497	1.483	1.562
		-0.015	0.877	-3.238	-1.827	1.132	-0.543	1.466	1.573
		0.037	1.050	-3.485	-1.938	1.104	-0.502	1.587	1.608
Average of ΔΔCt for *flhDC*		-1.184e-15	1.124	-3.376	-1.889	1.114	-0.514	1.512	1.581
Standard deviation of ΔΔCt for *flhDC*		0.027	0.238	0.103	0.046	0.013	0.021	0.053	0.020
Relative gene expression level		1.000	0.459	10.379	3.704	0.462	1.428	0.351	0.334
Standard deviation for relative gene expression level		0.018	0.076	0.740	0.118	0.004	0.020	0.013	0.005
